# Supplementary material for: Enrichment of Mango Fruit Leathers with Natal Plum (Carissa macrocarpa) Improves Their Phytochemical Content and Antioxidant Properties
Source: Foods. 2020 Apr 4;9(4):431. doi: 10.3390/foods9040431 (PMC7230645; doi:10.3390/foods9040431)
Supplement: Supplementary file 1 [file foods-09-00431-s001.pdf]

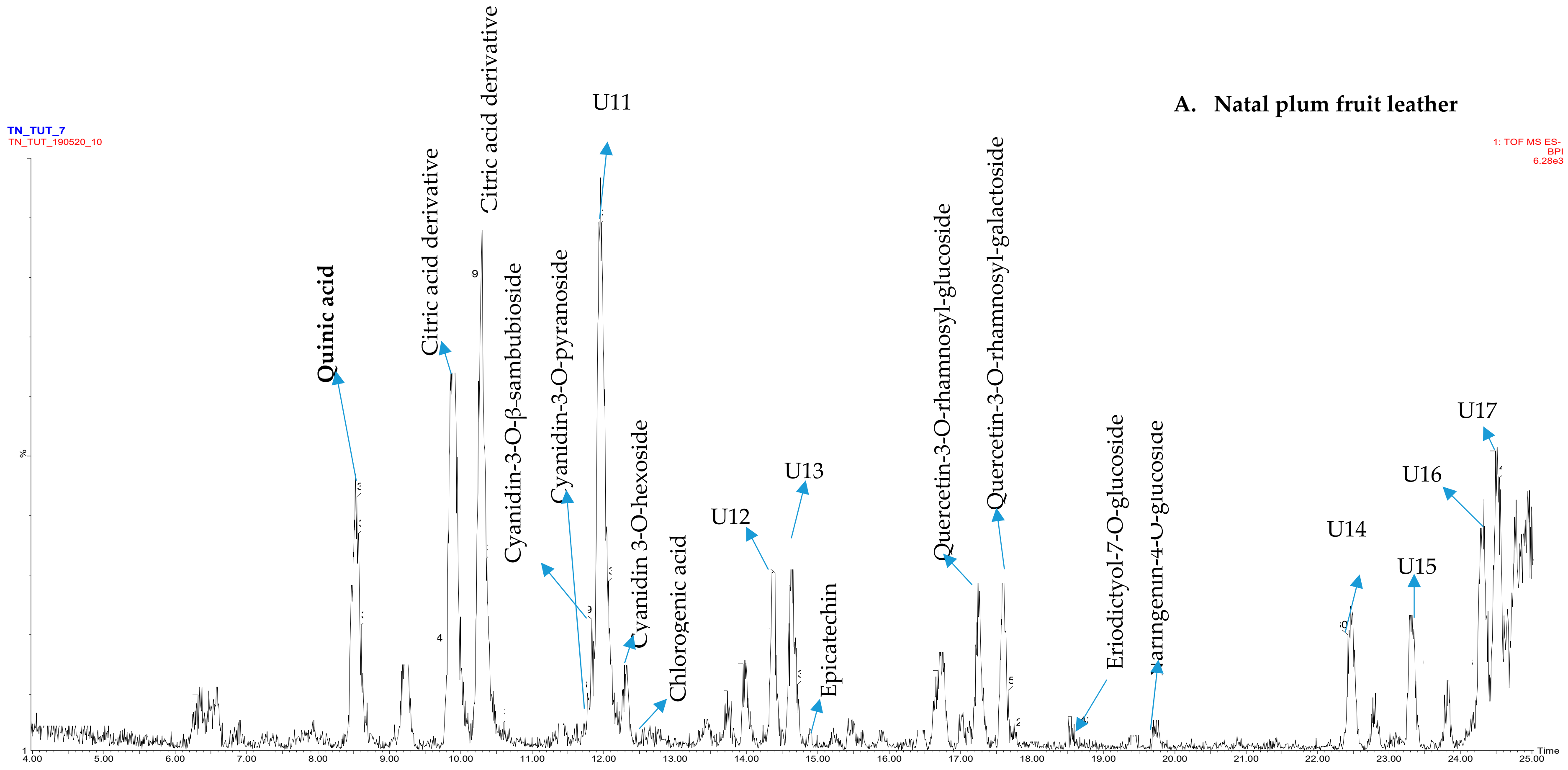
A. Natal plum fruit leather
TN_TUT_7
TN_TUT_190520_10
1: TOF MS ES-
BPI
6.28e3
Quinic acid
Citric acid derivative
Citric acid derivative
Cyanidin-3-O-β-sambubioside
Cyanidin-3-O-pyranoside
U11
Cyanidin 3-O-hexoside
Chlorogenic acid
U12
U13
Epicatechin
Quercetin-3-O-rhamnosyl-glucoside
Quercetin-3-O-rhamnosyl-galactoside
Eriodictyol-7-O-glucoside
Naringenin-4-O-glucoside
U14
U15
U16
U17
%
Time
4.00
5.00
6.00
7.00
8.00
9.00
10.00
11.00
12.00
13.00
14.00
15.00
16.00
17.00
18.00
19.00
20.00
21.00
22.00
23.00
24.00
25.00

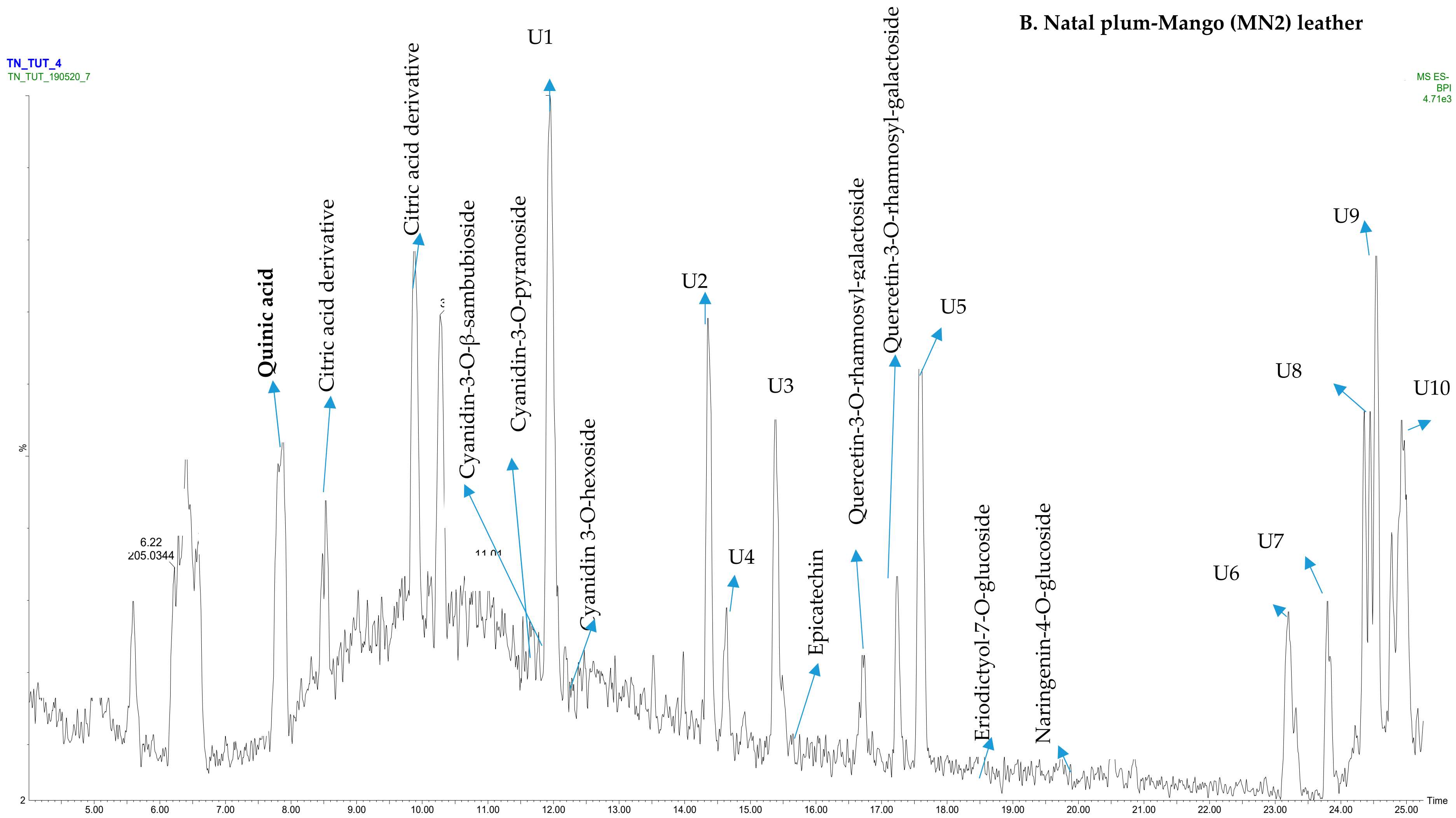

Figure S1. Comparison of UPLC–Q-TOF/MS chromatogram illustrating the changes in major phenolic compounds and organic acids in (**A**) Natal plum–Mango fruit leather in comparison to (**B**) Natal plum fruit leather. The chromatograms of three replicates of each treatment were included. The relative peak intensity is normalized, and peaks are expressed as the percentage highest peak intensity. U1 to U17—Unidentified compounds.
